# Optical Coherence Tomography Findings of Underlying Choroidal Neovascularization in Punctate Inner Choroidopathy

**DOI:** 10.3389/fmed.2021.758370

**Published:** 2021-12-22

**Authors:** Aniruddha Agarwal, Sabia Handa, Alessandro Marchese, Salvatore Parrulli, Alessandro Invernizzi, Roel J. Erckens, Tos T. J. M. Berendschot, C. A. B. Webers, Reema Bansal, Vishali Gupta

**Affiliations:** ^1^Advanced Eye Center, Post Graduate Institute of Medical Education and Research, Chandigarh, India; ^2^Cleveland Clinic, Eye Institute, Abu Dhabi, United Arab Emirates; ^3^Department of Ophthalmology, Istituto di Ricovero e Cura a Carattere Scientifico Ospedale San Raffaele, San Raffaele Scientific Institute, Vita-Salute San Raffaele University, Milan, Italy; ^4^Department of Biomedical and Clinical Science “Luigi Sacco,” Eye Clinic, Luigi Sacco Hospital, University of Milan, Milan, Italy; ^5^Save Sight Institute, University of Sydney, Sydney, NSW, Australia; ^6^Maastricht University Medical Centre+, University Eye Clinic Maastricht, Maastricht, Netherlands

**Keywords:** punctate inner choroidopathy, optical coherence tomography, choroidal neovascularization, optical coherence tomography angiography, uveitis, imaging

## Abstract

**Purpose:** To analyze findings on optical coherence tomography (OCT) suggestive of choroidal neovascularization (CNV) in lesions of punctate inner choroidopathy (PIC).

**Methods:** In this multi-center retrospective study, clinical data of patients with PIC were retrospectively analyzed. Quantitative data (height, width, and volume of PIC lesions), and qualitative data (disruption of ellipsoid zone (EZ)/Bruch's membrane (BM), outer retinal fuzziness, and choroidal back-shadowing) were compared between CNV+ and CNV– groups using Mann–Whitney *U*-test and Fischer's exact test.

**Results:** In total, 35 eyes (29 patients; 21 women; mean age: 33.3 ± 6.5 years) were selected for analysis. Of the 35 PIC lesions studied, 17 had underlying CNV. Lesions with CNV+ had larger height, width, and volume (*p* < 0.001) and several distinctive features, such as disruption of EZ and BM, outer retinal fuzziness, and hypo-reflective back-shadowing (*p* < 0.001) compared with CNV—lesions.

**Conclusions:** Quantitative and qualitative OCT analysis can aid in the prediction of an underlying CNV in the eyes with PIC.

## Introduction

Punctate inner choroidopathy (PIC) is a rare form of posterior uveitis characterized by multifocal yellow-white choroidal lesions that appear punctate, discrete, and well-demarcated. Most commonly, PIC is encountered in young myopic women (1–3). The presence of choroidal neovascularization (CNV) can be as high as 75% in eyes with PIC, making the detection of an underlying CNV critical in the management of this disease (4–7).

The inflammatory lesions of PIC have been evaluated using spectral-domain optical coherence tomography (SD-OCT) by Zarranz-Ventura et al. (8). The authors described three patterns of OCT in patients with PIC, namely atrophic pattern with outer retinal disruption (47%), sub-retinal pigment epithelial (RPE) cell deposit (34%), and localized RPE elevation (19%). The latter two patterns present either with sub-RPE hyper-reflective material or subretinal hyper-reflective material (SHRM). These patterns are relevant because an underlying CNV, which is very common in untreated eyes of PIC, can present similarly.

In this regard, Astroz et al. (9) have demonstrated the utility of OCT angiography (OCTA) in differentiating inflammatory multifocal choroiditis lesions from CNV lesions. In their study, OCTA was able to identify CNV in 14% of the cases that were undetected on other multimodal imaging (9). Similarly, Pohlmann et al. (5) demonstrated the utility of OCTA in detecting CNV lesions in PIC. While OCTA and dye-based angiographies, such as fluorescein angiography (FA) and indocyanine green angiography (ICGA) are highly sensitive in detecting CNV lesions (5, 10–12), it is important to revisit the OCT findings and assess whether OCT line scans show differences in eyes that harbor an underlying CNV from those which do not. In this study, we compared the OCT findings of eyes diagnosed with PIC with and without CNV lesions.

## Materials and Methods

### Study Subjects and Inclusion Criteria

This multicenter cross-sectional study was undertaken after approval from the Institutional Ethics Committee (IEC) of three tertiary-level uveitis referral hospitals: Post Graduate Institute of Medical Education and Research (PGIMER), Chandigarh, India; Luigi Sacco Hospital, University of Milan, Italy; and San Raffaele University, Milan, Italy. This study adheres to the declaration of Tenets of Helsinki.

The recently published Standardized Uveitis Nomenclature (SUN) working group provides the criteria for the diagnosis of PIC (13). The diagnosis of PIC was established based on clinical features of the typical disease, which consist of multifocal, small (1/4–1/2 disc diameter, <250 μm), discrete, yellow-white punctate choroidal inflammatory lesions confined to the posterior pole without overlying vitritis or anterior chamber inflammation (2, 8). Patients with atypical PIC lesions were excluded (8, 14). Subjects were included once infective etiologies and other causes of choroiditis, such as tuberculosis were ruled out. The other exclusion criteria were positive serology for syphilis (using a treponemal test, such as fluorescent treponemal antibody absorption test), and evidence of sarcoidosis due to the presence of bilateral hilar lymphadenopathy on imaging of the chest, or biopsy showing non-caseating granulomas.

Clinical and demographic data obtained from all the subjects, such as age, gender, refraction, treatment history (e.g., intravitreal injections), investigations, laterality, best-corrected visual acuity (BCVA) measured on Snellen's visual acuity chart (converted to LogMAR unit for analysis), and total follow-up duration. Details of the slit-lamp and fundus examination were noted for all patients. Patients with <6 months of follow-up duration were excluded from the analysis. In addition, eyes with media opacity (due to small pupil, cataract, or other reasons) and poor fixation precluding adequate imaging quality were excluded.

### Image Acquisition

Fundus imaging was done with Visupac FF450, Carl Zeiss Meditec, Jena, Germany, SD-OCT using Spectralis®, Heidelberg Engineering, Heidelberg, Germany, and swept-source (SS)-OCTA using DRI Triton®, Topcon, Japan. Combined FA and ICGA were done using HRA Spectralis®. The OCT scans were obtained using the dense raster scan protocol taking 20° × 20° scan centered through the fovea. Each individual B-scan was obtained after a minimum of 50 averaged scans. Customized dense scan protocols were used in case the lesion of interest was at a different location in the macula. The accompanying near infrared image was obtained with the OCT. A similar protocol was used in all three centers. SS-OCTA was performed using the 3 × 3 mm protocol centered on the fovea. The imaging data were collected by a data operator at each center who was responsible for data handling, indexing, and maintenance. Only those PIC lesions present in the central 3 × 3 mm were chosen for further analysis since they could be studied on both OCTA and OCT imaging.

### Image Analysis

The OCT image analysis was performed in a retrospective manner. First, the patients were evaluated clinically, image acquisition was done using fundus photography, OCT, and OCTA. The presence of CNV was confirmed on OCTA, and the patients were treated based on these findings. OCT image analysis has been described below:

### Quantitative Analysis

**Figure 1:**
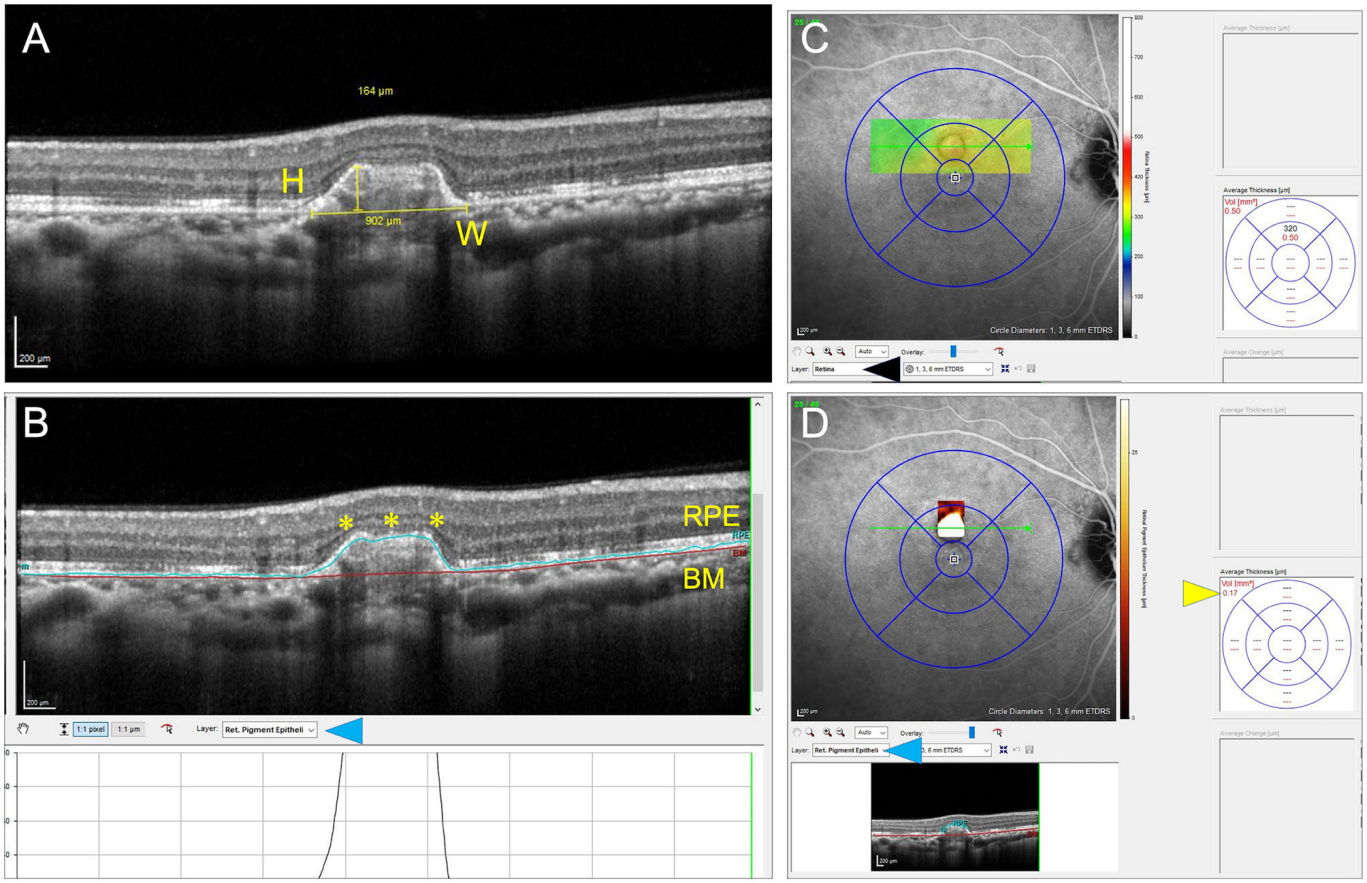
This figure explains the process of measuring the quantitative metrics of the lesions of punctate inner choroidopathy (PIC). The height (H) and width (W) measurements of the inflammatory PIC lesion are shown in **(A)**. **(B)** Shows the measurement of the volume of the PIC lesion after performing manual segmentation of the retinal pigment epithelium (RPE) line and correcting the Bruch's membrane (BM) line. The corrected RPE line is highlighted by yellow asterisks. The layer segmentation selection is shown by a blue arrowhead. **(C,D)** Show the technique of obtaining the volume measurements. By default, the software shows the volume of the entire retinal scan because the layer selection is “retina” (black arrowhead). When the RPE layer is selected (blue arrowhead), the volume of the RPE lesion is obtained (yellow arrowhead) **(D)**.

Using the color fundus photographs, and near infra-red images, PIC lesions in each eye in the central 3 × 3 mm were counted for inclusion. Lesions that were either sub-RPE deposits or localized RPE elevation with SHRM based on the classification by Zarranz-Ventura et al. (8) were included. Thus, atrophic lesions with outer retinal disruption (which are predominant in treated eyes) were excluded from the analysis. Each PIC lesion was carefully quantified on SD-OCT imaging in terms of its height, width, and volume using the Heyex® software (version 6.1). For measuring the height of the PIC lesion, the highest elevation of the lesion from the Bruch's membrane (BM) was identified on consecutive OCT raster scans and measured using the linear caliper tool on Heyex (in microns). On the same scan used for measuring the height, we measured the width with the linear caliper tool at the base of the dome (along with the BM) (in microns). All the individual OCT scans encompassing the PIC lesion were then manually re-segmented on Heyex (on the thickness profile tab) so that the “RPE” layer was drawn along the increased dome of the PIC lesion, and the “BM” layer was along with the BM. Using the quantitative parameters available on the thickness map tab, the “RPE layer” was chosen on the Early Treatment Diabetic Retinopathy Study (ETDRS) circle. This selection provides the volume of the PIC lesion along with the average thickness on the ETDRS grid. Figure 1 shows a diagrammatic representation of obtaining the quantitative metrics.

All the quantitative measurements on OCT were performed by two graders (AA and SH; both vitreoretinal and uveitis specialists). The average of the two graders was chosen for the analysis.

### Qualitative Analysis

The included lesions of PIC were evaluated for the presence of CNV using multimodal imaging, i.e., FA, ICGA, and OCTA. The clinical notes and treatment records were reviewed and correlated with the imaging findings. The presence of CNV was identified based on the leakage on FA and the appearance of a vascular network on ICGA. OCTA scans were carefully segmented to see any visible neovascular network and abnormal flow signal on the cross-sectional OCT B-scan. The PIC lesions were classified as those with CNV **(CNV+)** and those without **(CNV–)**. Each PIC lesion was evaluated for the following qualitative features on SD-OCT:

a. RPE elevation: defined as the focal elevation of RPE with a clear space between the RPE and the underlying BM
b. Disruption of the ellipsoid zone of the photoreceptors
c. Disruption of the BM
d. Involvement of the outer retina: defined as fuzzy infiltration of the outer retinal layers, specifically the outer plexiform layer
e. Presence of either intra- or subretinal fluid
f. Hyper- or hypo-reflective back-shadowing beneath the RPE in the choroid

**Figure 2:**
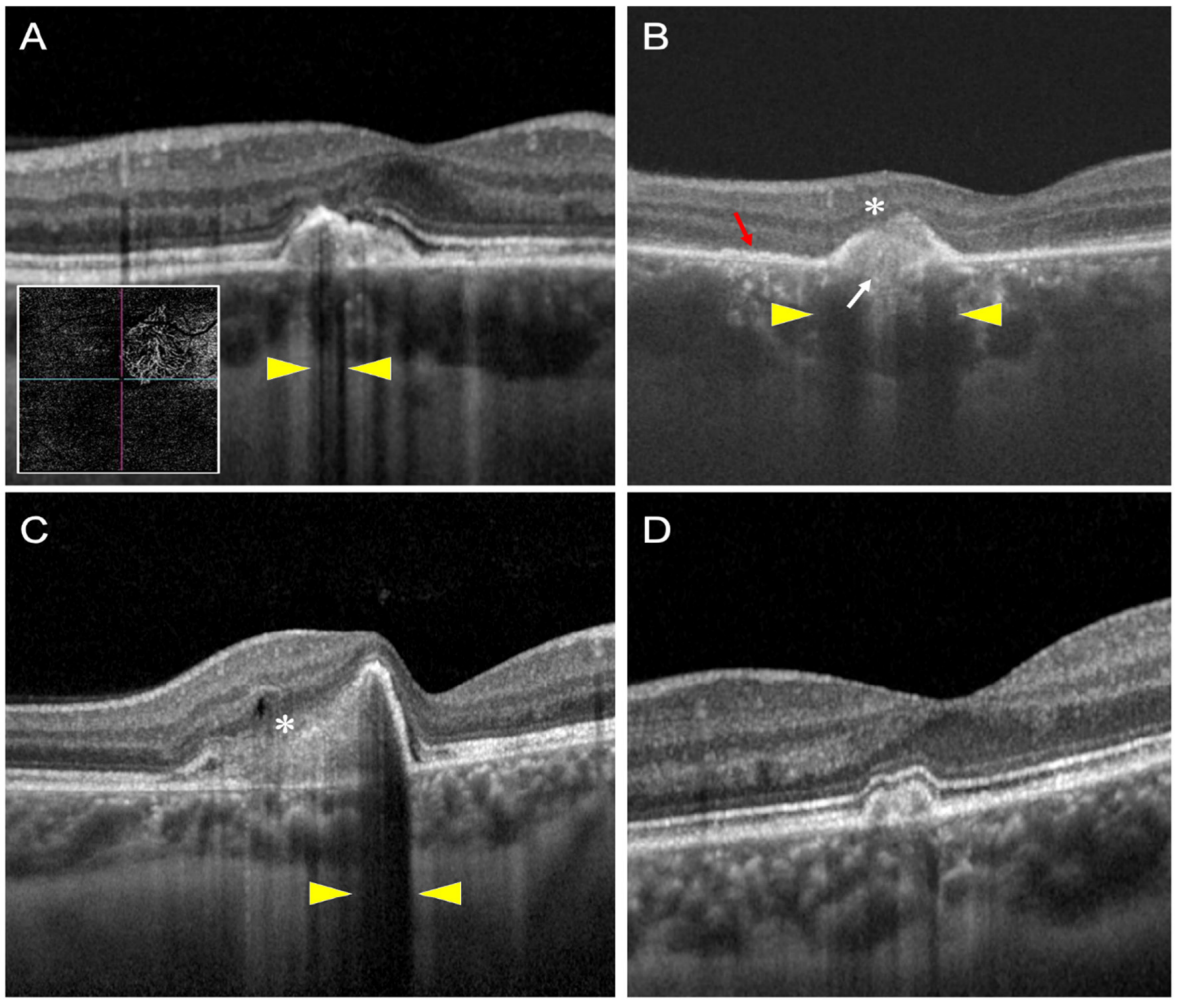
This figure shows examples of optical coherence tomography (OCT) scans of patients with PIC with and without choroidal neovascularization (CNV). **(A–C)** Show three patients with CNV, and **(D)** shows a patient without an underlying CNV. The OCT scan **(A)** shows hypo-reflective choroidal back-shadowing (yellow arrowheads). The inset shows an OCT angiography image with a large CNV network. OCT scan **(B)** shows a patient with outer retinal fuzziness (white asterisk), disruption of the ellipsoid zone (red arrow), disruption of the BM (white arrow), and hypo-reflective choroidal back-shadowing (yellow arrowheads). OCT scan **(C)** shows a third patient with fuzzy infiltration of the outer retina (white asterisk), a small intraretinal cystoid space, and hypo-reflective back-shadowing (yellow arrowheads). OCT scan of another patient with no underlying CNV shows none of these features.

The example of each pathological alteration is described in Figure 2.

### Statistical Analysis

Data analysis was performed using IBM SPSS Statistics® (IBM, Armonk, NY, USA) version 27.0. The normality of quantitative data was assessed using histograms. The quantitative data on OCT, such as height, width, and volume of PIC lesion were compared between the CNV+ and CNV– group using the Mann–Whitney U-test. The categorical data including the qualitative features, such as photoreceptor layer disruption and presence of fluid were compared between the two groups using Fischer's exact test. Data are expressed in mean and SD and *p* < 0.05 were considered statistically significant.

## Results

We included 35 eyes of 29 patients (8 men and 21 women), having a mean age of 33.2 ± 6.2 years and of which 19 were Caucasian and 10 were Asian Indian in ethnicity. All subjects were diagnosed with PIC based on the diagnostic criteria mentioned previously. Mean follow-up was 22.8 ± 7.4 months. Demographic characteristics and ongoing therapies are described in Tables 1, 2.

**Table 1:** Demographic and clinical characteristics of patients with punctate inner choroidopathy (PIC) included in the study.

**Variable**		**All patients**	**CNV+**	**CNV-**	***p*** **-value**
Number of patients (n)		29	15	14	
Ethnicity	Caucasian (n)	19	9	10	0.80
	Asian Indian (n)	10	6	4	
Age (years ± stdev)		33.3 ± 6.5	31.8 ± 5.0	35.0 ± 7.6	0.19
Gender	Male (n)	8	2	6	0.11
	Female (n)	21	13	8	
Follow-up (months)		22.9 ± 7.3	23.2 ± 6.7	22.6 ± 8.1	0.82
Systemic corticosteroids alone (n)		13	8	5	0.36
Oral azathioprine (n)		8	2	6	
Oral mycophenolate mofetil (n)		5	3	2	
None (n)		3	2	1	

**Table 2:** Characteristics of eyes with PIC included in the study.

**Variable**	**All eyes**	**CNV+**	**CNV-**	***p*** **-value**
Number of eyes	35	17	18	
Initial BCVA (LogMAR)	0.29 ± 0.28	0.38 ± 0.35	0.22 ± 0.18	0.28
Final BCVA (LogMAR)	0.23 ± 0.21	0.35 ± 0.24	0.13 ± 0.11	**0.002**
Intravitreal ranibizumab (eyes) Number of injections		10 3.6 ± 1.9	0	
Intravitreal bevacizumab (eyes) Number of injections		7 4.6 ± 1.5	0	

*The values indicated in bold are statistically significant*.

In the central 3 × 3 mm region imaged by the OCTA, a single PIC lesion was captured in 33 eyes. In the remaining 2 eyes, 2 lesions were captured. However, only a single lesion was chosen randomly in these eyes (so that one lesion per eye was analyzed). Among the 35 lesions analyzed, 17 lesions were categorized as CNV+ based on FA, ICGA, and OCTA imaging characteristics. There were no differences in age and gender between patients with and without CNV (*p* = 0.19 and 0.11, respectively) (as shown in Table 1). In addition, we found no differences in initial BCVA between eyes with CNV and without CNV (0.38 ± 0.35 vs. 0.22 ± 0.17, *p* = 0.28). However, the final BCVA at the end of the follow-up was significantly lower among eyes with CNV compared with those without CNV (0.35 ± 0.24 vs. 0.13 ± 0.11; *p* = 0.002) (as shown in Table 2).

Table 3, summarizing the quantitative analysis of the PIC lesions on OCT imaging, shows that those with CNV+ had a larger volume, height, and width compared with lesions without CNV. Figure 2 shows a representation comparing CNV+ and CNV– lesions along with the measurements.

**Table 3:** Quantitative analysis of lesions of PIC on spectral domain optical coherence tomography (SDOCT).

**Variable (micron)**	**All lesions**	**CNV+**	**CNV-**	***p*** **-value** ^*^
Number of lesions	35	17	18	
Volume	0.30 ± 0.25	0.44 ± 0.27	0.17 ± 0.12	**0.001**
Height	156 ± 81	184 ± 80	129 ± 76	**0.02**
Width	1,315 ± 688	1,705 ± 587	947 ± 560	**<0.001**

* *p-values were calculated using Mann–Whitney U-test. The values indicated in bold are statistically significant*.

Table 4 summarizes the qualitative analyses of the PIC lesions. Significantly larger number of CNV+ lesions showed focal RPE elevation compared with CNV– lesions (*p* = 0.001). Disruption of the ellipsoid zone/photoreceptors and BM was significantly higher in CNV+ lesions compared with CNV–lesions (*p* = < 0.001). Fuzzy infiltration of the outer retina was seen in significantly higher CNV+ lesions (*p* < 0.001). Ten CNV+ lesions had associated intra- or subretinal fluid on OCT compared with none with CNV– (*p* < 0.001). Among lesions with CNV+, there were significantly higher lesions with hypo-reflective back-shadowing beneath the RPE compared with CNV– lesions (*p* < 0.001). CNV– lesions showed hyper-transmission of the OCT signal beneath the RPE into the choroid.

**Table 4:** Qualitative features PIC lesions on SDOCT.

**Variable**	**All lesions**	**CNV+**	**CNV-**	***p*** **-value** ^*^
Number of lesions	35	17	18	
Focal RPE elevation	28	17	10	**0.001**
Disruption of ellipsoid zone and photoreceptors	22	15	6	**0.001**
Disruption of Bruch's membrane	18	16	2	**<0.001**
Fuzzy infiltration of outer retina	13	13	0	**<0.001**
Intra- or subretinal fluid	10	10	0	**<0.001**
**Back-shadowing beneath RPE**				
Hypo/Iso-reflectivity	17	16	1	**<0.001**
Hyper-reflectivity	20	1	17	

* *p-values were calculated using Fischer's exact test. The values indicated in bold are statistically significant*.

For the analysis in Table 3, we assumed all lesions to be independent. However, for some subjects, two eyes were included. Therefore, we repeated the analysis for left and right eyes separately. For both eyes, the differences between CNV+ and CNV– were similar as shown in Table 3 and significant for volume and height. For the height of the lesions, we found a *p*-value of 0.19 and 0.10 for the left and right eye, respectively. Additionally, we repeated the analysis leading to Table 4 for both eyes separately. Again, we found significant results pointing in the same direction in both eyes.

We did not find a relation between the morphological features on one hand and ethnicity, age, gender, or therapy on the other hand.

## Discussion

A significant proportion of patients with PIC and multifocal choroiditis are at high risk of developing significant vision loss due to the development of CNV (5, 6, 11, 12, 15–18). Over the years, OCTA has shown advantages in non-invasive imaging of CNV in various conditions, such as age-related macular degeneration (AMD), myopia, and uveitis (12, 15, 19–23). In AMD, the detection of subclinical non-exudative CNV has changed the classification of the disease enabling the identification of population at risk of severe vision loss (24, 25). While OCTA has gained widespread acceptability in the clinical assessment of patients with retinal diseases and uveitis, OCT imaging remains a cornerstone in their management. Our results of OCT analyses of patients with PIC reveals various findings that can provide valuable insights before embarking upon advanced (and more expensive) tools, such as OCTA, and invasive tests, such as FA.

The analysis of PIC lesions in our series revealed that those with an underlying CNV were significantly larger on OCT imaging. The CNV+ lesions had a significantly larger volume, height, and width compared with CNV– lesions. Of note, the lesions with CNV had nearly two times the width of CNV– lesions (~1,700 vs. 950 μm; Table 3). This seems to be a feature that can readily differentiate lesions harboring an underlying CNV compared with those which do not. These quantitative metrics are in line with the findings from eyes with AMD. Greater SHRM height and width on OCT in eyes with AMD have been correlated with poorer visual acuity due to the presence of an underlying CNV (26). This has practical relevance because deep learning algorithms have already been applied in AMD to quantify SHRM parameters (27). Thus, in the future, quantitative metrics of SHRM may have relevance in non-AMD conditions, such as uveitis.

Our analyses revealed certain novel findings on OCT that strongly suggest the presence of an underlying CNV. These features are summarized in Table 4. The CNV+ lesions had a significantly higher prevalence of focal RPE elevations with a clear space separating them from the BM. The disruption of photoreceptors and BM were important features that differentiated CNV+ from CNV– lesions. None of the eyes with CNV– lesions had fuzzy infiltration involving the outer retina. This feature may serve as an important biomarker of an underlying CNV on OCT in PIC (Figure 2).

The presence of CNV resulted in hypo-reflective back-shadowing in the choroid in the area of the lesion. The hypo-reflective back-shadowing has been previously described as a marker of an underlying CNV by Shi et al. (28). The authors analyzed 22 eyes with myopia and divided the patients into two groups: those with myopic CNV (*n* = 10) and those with PIC but no CNV (*n* = 12). In this study, 100% of eyes with myopic CNV and eyes developing CNV during the course of the disease showed choroidal hypo-transmission below the SHRM. On the other hand, the inflammatory PIC lesions without CNV showed either iso-transmission or hyper-transmission, similar to our findings. Besides Shi et al. other authors, such as Amer et al. (29) have described the relevance of OCT imaging in differentiating inflammatory CNV from acute inflammatory lesions. A potential reason for choroidal hypo-reflectivity beneath the CNV could be the infiltration of metabolically active CNV resulting in back-shadowing. Thus, our findings reiterate the importance of choroidal reflectance in PIC on OCT.

Recently, the SUN working group has elucidated the new classification criteria of PIC using a robust 4-phase study consisting of machine learning using the patient dataset from over 75 investigators worldwide. Analysis of 250 patients, the study identified key features of PIC, such as characteristic punctate choroidal lesions, absent anterior/vitreous inflammation, and posterior pole involvement (13). OCT staging of PIC lesions has been described previously by Zhang et al. (using spectral-domain OCT) (30) and Zarranz-Ventura et al. (8) (using enhanced-depth imaging OCT). Both the staging systems proposed progressive inner retinal infiltration up to the outer plexiform layer, followed by progressive outer retinal, RPE, and choroidal atrophy. Both the staging systems did not consider the presence of an underlying CNV, which frequently develops in the eyes with PIC. However, to be consistent with the known attributes of PIC on OCT, we excluded eyes with atypical lesions and included only those lesions which are presented with localized RPE elevation with SHRM.

Inflammatory CNV can be affected by ongoing immunosuppressive therapies and patient demographic factors, such as age, gender, and ethnicity (15). In our series, both groups, i.e., CNV+ and CNV–, were statistically similar for various features, such as age, ethnicity, gender, and ongoing treatments (Table 1). This has an advantage as it can minimize the potential selection bias.

The exact pathogenesis of inflammatory CNV in PIC and other uveitic conditions is still unknown, but the final common pathway includes disruption of the RPE–Bruch's complex and a vascular endothelial growth factor (VEGF) drive resulting in aberrant angiogenesis (2, 3, 15, 21). A revised in-depth analysis of the disease using imaging tools, such as OCTA, OCT, and dye-based angiographies can help improve our understanding of the pathology, thereby improving the patient outcomes in the future.

Limitations of this study are the limited sample size and its cross-sectional design. The image analysis was performed in a retrospective manner. However, the selection criteria ensured that the two groups (CNV+ and CNV–) had no differences in age, gender, ethnicity, and ongoing immunosuppressive/corticosteroid therapies. None of the subjects had other concomitant diseases, such as macular scarring that could affect the assessments, such as SHRM dimensions. A longitudinal study of PIC lesions can provide further insights into the evolution of the inflammatory lesions and the risk factors for the development of CNV. In addition, automated quantitative analysis can aid in the rapid evaluation of the lesions in the future.

In summary, PIC lesions harboring an underlying CNV can have distinctive features on OCT imaging that help differentiate them from acute inflammatory lesions with no underlying CNV. Lesions harboring CNV tend to be larger in dimensions (especially width) on OCT and have a higher prevalence of fuzzy infiltration of the outer retina, disruption of the ellipsoid and photoreceptor zone, and choroidal hypo-reflectivity and back-shadowing.

## Data Availability Statement

The raw data supporting the conclusions of this article will be made available by the authors without undue reservation.

## Ethics Statement

The studies involving human participants were reviewed and approved by Institute Ethics Committee (IEC) of Post Graduate Institute of Medical Education and Research (PGIMER), Chandigarh, India. Written informed consent for participation was not required for this study in accordance with the national legislation and the institutional requirements.

## Author Contributions

AA and SH conceptualized the manuscript and drafted the manuscript. SH, AM, SP, and AI collected all the data. The interpretation and data analysis was done by AA, AM, SH, AI, RE, TB, and CW. RE, TB, CW, RB, and VG provided critical inputs and helped in drafting the manuscript. AM, SP, AI, RE, TB, CW, RB, and VG finalized the manuscript for submission. All authors contributed to the article and approved the submitted version.

## Conflict of Interest

The authors declare that the research was conducted in the absence of any commercial or financial relationships that could be construed as a potential conflict of interest.

## Publisher's Note

All claims expressed in this article are solely those of the authors and do not necessarily represent those of their affiliated organizations, or those of the publisher, the editors and the reviewers. Any product that may be evaluated in this article, or claim that may be made by its manufacturer, is not guaranteed or endorsed by the publisher.
